# Microsurgical resection of symptomatic intramedullary cervical spinal cord cavernous malformation

**DOI:** 10.3171/2019.7.FocusVid.19137

**Published:** 2019-07-01

**Authors:** Tomasz A. Dziedzic, Andrzej Marchel

**Affiliations:** Department of Neurosurgery, Medical University of Warsaw, Warsaw, Poland

**Keywords:** cavernous malformation, spinal cord, intramedullary cavernoma, spinal vascular malformation, video

## Abstract

Intramedullary cavernous malformations account for approximately 5% of all intraspinal lesions. Symptomatic lesions are treated with microsurgical resection. Due to surrounding eloquent spinal neural tissue, surgical removal of these lesions can be technically challenging. Surgical treatment carries a significant risk for postoperative morbidity. This video demonstrates the main steps for the microsurgical technique of resection of a symptomatic intramedullary cervical spinal cord cavernous malformation at the C2–3 level. Complete resection was achieved with minimal posterior column deficit. The operative technique and surgical nuances, including the patient’s positioning, surgical approach, intraspinal cavernous malformation removal, and closure, are illustrated.

The video can be found here: https://youtu.be/UKttTiXlEb8.

## Transcript

This video will demonstrate the microsurgical technique for resection of an intramedullary cervical spinal cord cavernous malformation.

00:30 Clinical presentation

The patient is a 33-year-old who presented with progressive and recurrent sensory symptoms. He complained of chronic neck pain and recurrent right upper extremity paresthesia. Neurological examination on admission demonstrated normal strength in all extremities, with sensory disturbance within the right upper extremity.

00:53 Imaging

Preoperative cervical MRI shows a midline and dorsally located mixed-signal-intensity lesion of popcorn-like appearance which is characteristic for intraspinal cavernous malformation at the C2–3 level. Additionally, hemosiderin deposits around lesion are visualized.

Due to recurrent symptoms in a young patient, and the superficially located lesion combined with the potential for severe neurological deficit due to another hemorrhage, a microsurgical resection was performed.

01:30 Positioning

For posterior approach to the upper cervical spine we prefer semisitting position which provides gravitational drainage of blood and thanks to that clean operating field. In our experience position-related morbidity in semisitting position is comparable to other forms of positioning when dorsal cervical spine is operated.

01:55 Exposure

The spine is exposed through a midline incision and subperiosteal muscle dissection. A C2–3 laminectomy is performed.

After dural exposure the dura is opened in the midline and dural leaflets are tucked up with sutures and the intact arachnoid is exposed. The same is done with arachnoid which is mobilized off the spinal cord and tucked up with dural clips. The cavernous malformation can be seen now on dorsal surface in the midline. Cavernoma has a characteristic appearance as demonstrated in this case.

02:25 Myelotomy

The incision of the spinal pia is performed close to midline over the cavernoma. Usually, there are small brisk bleeding which usually stops with warm saline irrigation. Electrocautery is avoided when possible.

Then the plane between the cavernoma and spinal cord is found and the pial tuck-up sutures can be placed as in this case. The reddish cavernoma is dissected away from the gliotic plane which is usually yellowish due to hemosiderin deposits.

02:50 Dissection

Surgical plane between cavernoma and spinal cord is developed with gentle traction and countertraction technique. Progressive circumferential detachment of the cavernoma continues. Fibrous, thickened adhesions and feeding vessels are isolated, cauterized with bipolar cautery, and divided with microscissors; this is followed with blunt dissection. The plane is developed all around the lesion.

Gentle bipolar cautery on the cavernoma surface can also effectively shrink cavernoma mass, which makes it easier to manipulate on it. Around the whole lesion traction-countertraction technique followed by bipolar cauterization of vessels sharp and blunt dissection is applied. The lesion can be retracted and dissected away from the spinal cord with gentle traction, which can be improved with a cottonoid placed on the lesion.

The extralesional dissection with blunt instrument is continued around the lesion and again small current bipolar forceps are used to better visualize the plane and to shrink the lesion. Further blunt dissection carried around the lateral margins of the cavernoma leads us to the ventral side of the lesion.

Development of the deep cavernoma spinal cord interference precedes circumferentially at the cavernoma’s margins. Firstly, we identify the ventral margin on the lateral side of the cavernoma. Then the ventral surface is found from superior margin of the lesion, and finally the same is achieved from inferior.

The last adhesion point of the cavernoma is identified, coagulated, and cut with microscissors, which makes it ready to remove it in an unblock fashion.

06:00 Hemostasis

The resection bed is inspected under high magnification to ensure complete resection and secure hemostasis.

06:15 Dural closure

Meticulous hemostasis is achieved. Pial tuck-up sutures are released, and the edges of the dura are reapproximated and dura is closed in watertight fashion with a running 5-0 suture.

Standard multilayer soft-tissue wound closure is performed.

The patient did well and was ambulatory on the postoperative day number 1 with minimal posterior column deficit worse on the right side.

07:00 Postoperative imaging

MR obtained 3 months postoperatively demonstrates no residual cavernoma with hemosiderin deposits.
